# Aberrant Cx26 hemichannels and keratitis-ichthyosis-deafness syndrome: insights into syndromic hearing loss

**DOI:** 10.3389/fncel.2014.00354

**Published:** 2014-10-27

**Authors:** Helmuth A. Sanchez, Vytas K. Verselis

**Affiliations:** Dominick P. Purpura Department of Neuroscience, Albert Einstein College of MedicineBronx, NY, USA

**Keywords:** connexin, hemichannel, deafness, cochlea, gating, permeability, calcium

## Abstract

Mutation of the *GJB2* gene, which encodes the connexin 26 (Cx26) gap junction (GJ) protein, is the most common cause of hereditary, sensorineural hearing loss. Cx26 is not expressed in hair cells, but is widely expressed throughout the non-sensory epithelial cells of the cochlea. Most *GJB2* mutations produce non-syndromic deafness, but a subset produces syndromic deafness in which profound hearing loss is accompanied by a diverse array of infectious and neoplastic cutaneous disorders that can be fatal. Although GJ channels, which are assembled by the docking of two, so-called hemichannels (HCs), have been the main focus of deafness-associated disease models, it is now evident that the HCs themselves can function in the absence of docking and contribute to signaling across the cell membrane as a novel class of ion channel. A notable feature of syndromic deafness mutants is that the HCs exhibit aberrant behaviors providing a plausible basis for disease that is associated with excessive or altered contributions of Cx26 HCs that, in turn, lead to compromised cell integrity. Here we discuss some of the aberrant Cx26 HC properties that have been described for mutants associated with keratitis-ichthyosis-deafness (KID) syndrome, a particularly severe Cx26-associated syndrome, which shed light on genotype-phenotype relationships and causes underlying cochlear dysfunction.

## Cx26 mutations and deafness

It has been over 15 years since the *GJB2* gene encoding the Cx26 gap junction (GJ) protein was identified as a susceptibility gene for sensorineural deafness (Kelsell et al., 1997). In the original study, Cx26 mutations were shown to result in premature stop codons in several autosomal recessive non-syndromic deafness pedigrees. Cx26 mutations are now known to represent one of the most common causes of inherited, non-syndromic deafness in the human population (Apps et al., 2007; Chan et al., 2010; Duman and Tekin, 2012). More than 100 Cx26 mutations have been identified and, for the most part, they are recessive and produce loss of function as a result of deletions, insertions and frameshifts (Hoang Dinh et al., 2009; Lee and White, 2009). However, a subset of these mutations has been shown to cause sensorineural deafness that is accompanied by severe skin disorders. In contrast to non-syndromic deafness, syndromic deafness is characteristically cause by missense mutations that result in single amino acid substitutions and behave in an autosomal dominant manner.

**Figure 1:**
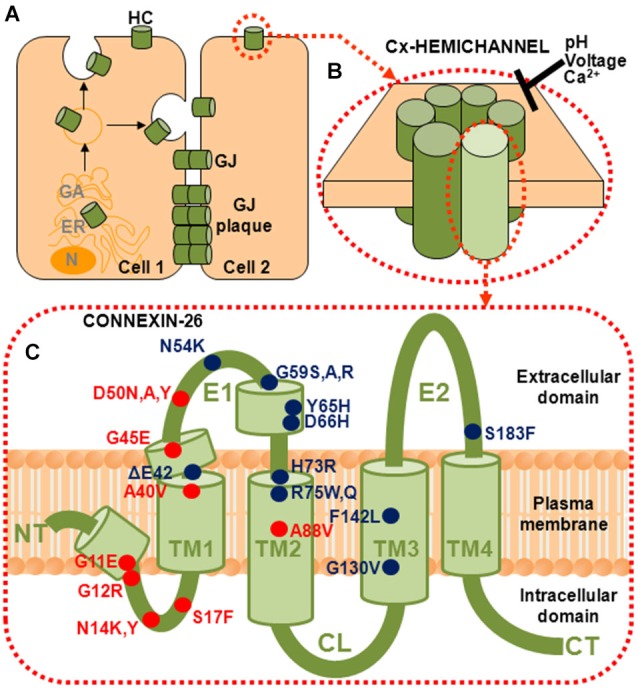
**Connexins (Cxs) form gap junction (GJ) channels and hemichannels (HCs). (A)** GJ channels are formed by of docking of two HCs from neighboring cells. GJ channels cluster into plaques and provide a direct intercellular communication pathway between cells. **(B)** HCs, also called connexons, can function in the plasma membrane in the absence of docking. They are hexamers of six Cx subunits and are strongly regulated by pH, voltage and divalent cations, principally Ca^2+^. **(C)** Topology of a Cx subunit. The amino (NT) and carboxy (CT) domains are oriented to the cytoplasm. The NT domain loops back into the membrane to contribute to the aqueous pore. There are four helical transmembrane domains (TM1–TM4) and two extracellular loop domains, E1 and E2. The short helical segments in NT and E1 identified in the crystal structure are also shown (Maeda et al., 2009). Locations of the 18 residues that have been associated with mutations causing syndromic deafness are indicated; those associated with KID syndrome are indicated in red.

To date, mutations at 18 positions in Cx26 have been identified in association with syndromic deafness (Figure 1). Based primarily on the nature of the cutaneous manifestations patients experience and the extent of ophthalmic involvement, mutations have been classified in one of several syndromes including keratitis-ichthyosis-deafness syndrome (KID), palmoplantar keratoderma (PPK), Bart-Pumphrey syndrome (BPS), Vohwinkel syndrome (VS) and hystrix-like ichthyosis deafness syndrome (HID; reviewed in Lee and White, 2009; Xu and Nicholson, 2013).

Cx channels are unique in that they can adopt two very different structural configurations, as intercellular GJ channels formed by the docking of two hemichannels (HCs), one contributed by each of two apposed cells, and as undocked HCs operating in the plasma membrane (Figures 1A,B). These two channel configurations perform very different functions, with GJs providing direct signaling between cells and HCs providing signaling across the plasma membrane. Given that loss of GJ function does not lead to skin disorders and that some syndromic deafness mutants function as GJs whereas others do not, the disease mechanisms in non-syndromic and syndromic cases likely differ. A property in common among syndromic deafness mutants is functional HCs that show aberrant properties, suggesting that aberrant signaling across the plasma membrane mediated by mutant HCs may be the principal cause of cellular dysfunction. For mutants that function as GJ channels and HCs, both channel configurations can contribute to disease mechanisms.

## Roles of GJs and HCs in the cochlea

**Figure 2:**
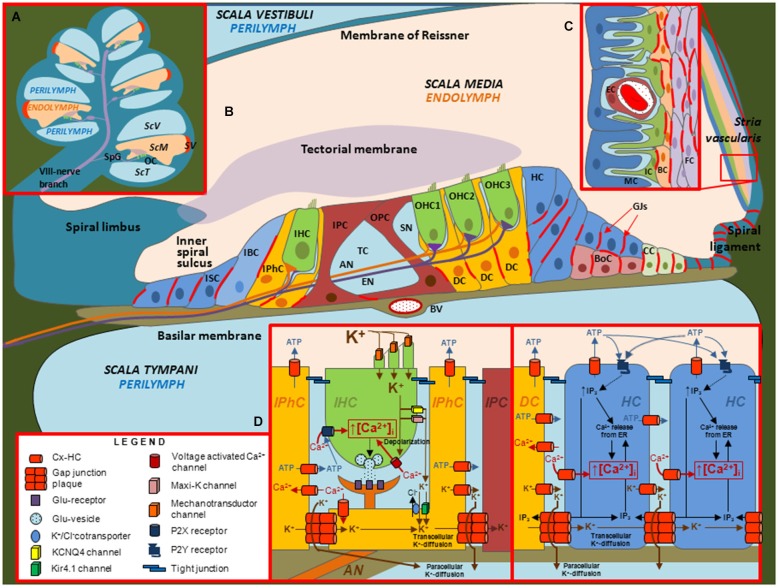
**Illustrations depicting the cellular organization of the mammalian cochlea and the putative roles of Cx channels. (A)** Transverse section through the cochlea. OC: organ of Corti. SpG: Spiral ganglia. *SV: stria vascularis. ScV: scala vestibuli. ScM: scala media. ScT: scala tympani*. **(B)** Diagram of the organ of Corti. In this representation, the relative sizes of the different structures are altered to emphasize the organ of Corti, which contains inner hair cells (IHC), outer hair cells (OHC), different types of support cells and structures including afferent (AN) and efferent (EN) nerve fibers, blood vessel (BV), Boettcher cells (BoC), Claudius cells (CC), Deiter’s cells (DC), Hensen cells (HC), Inner border cells (IBC), Inner pilar cells (IPC), Inner phalangeal cells (IPhC), Inner Sulcus cells (ISC), Outer pilar cells (OPC), space of Nuel (SN) and tunnel of Corti (TC). Inset **(C)** Structure of the *Stria vascularis* containing endothelial cells (EC), fibrocytes (FC), basal cells (BC), intermediate cells (IC) and marginal cells (MC). Inset **(D)** Putative roles of Cx channels in support cells. Cx HCs and GJ channels in support cells have been proposed to mediate the flux of K^+^, ATP, IP_3_ and Ca^2+^ across the membrane and between cells, respectively.

**Figure 3:**
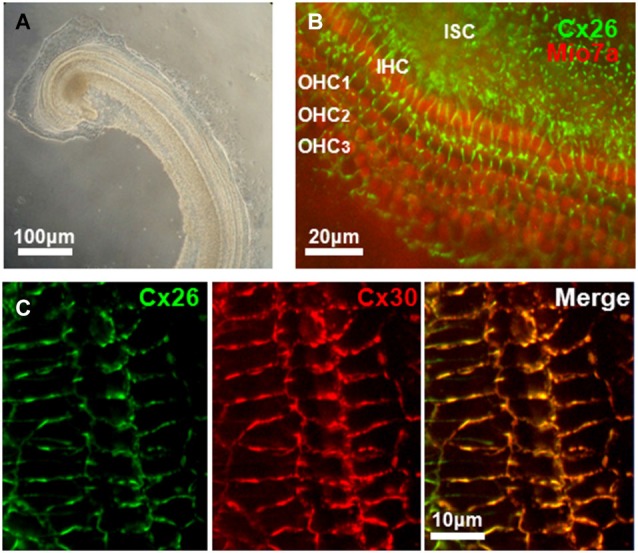
**Connexin expression in mouse cochlea. (A)** Low-power transmitted-light image of a primary explant culture of cochlea (72 h *in vitro*) prepared from a P4 mouse. **(B)** Immunoreactivity for Cx26 (green), and MyosinVIIa (red), marker of hair cells, obtained from an *in vitro* primary culture of mouse cochlea after 4 days in culture. Cx26 staining is widespread in cochlea support cells in regions of cell-cell contact. Inner (IHC) and outer (OHC) hair cells are indicated. ISC: Inner support cells. **(C)** Expression of Cx26 and Cx30 in support cells of organ of Corti in an *in vitro* primary culture of mouse cochlea after 5 days in culture. Merged image of Cx26 and Cx30 immunostaining shows extensive co-localization of both connexins.

Of the three fluid filled compartments in the cochlea, the scala media consisting of endolymph plays a special role in the transduction of electrical signals by sensory hair cells. The epithelium of the cochlea enclosing the endolymphatic compartment consists of sensory hair cells and surrounding support cells of the organ of Corti, Reissner’s membrane and the cells of the stria vascularis; the latter produces endolymph, which is high in K^+^ and low in Ca^2+^, and generates the endocochlear potential, EP (Figure 2). Two Cxs, Cx26 and Cx30, are expressed in the cochlea and immunostaining has shown expression of both Cxs is widespread in the support cells of the organ of Corti and in the stria vascularis; Cx expression is absent in hair cells (Lautermann et al., 1998; Ahmad et al., 2003; Forge et al., 2003; Zhao and Yu, 2006; Liu and Zhao, 2008). Figure 3 shows images of cultured cochlea explants from mice immunostained for Cx26 and Cx30. Regions of cell-cell contact show strong staining indicative of large clusters or plaques of GJ channels.

The expression patterns for Cx26 and Cx30 suggest that GJs contribute to the formation of extensive interconnected cellular networks and it has been proposed that these networks function as part of the recycling circuit for K^+^ between perilymph and endolymph (Lang et al., 2007; Zdebik et al., 2009; Adachi et al., 2013). K^+^ entering hair cells from the endolymph through mechano-sensitive channels in the apical surface exit at the basolateral membrane and are taken up by support cells via K^+^/Cl^−^ co-transporters and diffuse via GJs towards the lateral wall. The cells of the stria vascularis, via a series of transporters and K^+^ channels, produce movement of K^+^ back into the endolymph and the generation of the EP (Adachi et al., 2013), which is an important driving force for the influx of K^+^ that depolarizes hair cells during mechano-transduction. However, the availability of perilymphatic extracellular pathways for the movement of K^+^ to the fibrocytes (FC) of the spiral ligament, which are electrically coupled to the basal cells (BC) of stria vascularis (Liu and Zhao, 2008), has questioned the need for GJs in the support cells of the sensory epithelium for K^+^ recycling (Zdebik et al., 2009; Patuzzi, 2011). In addition, the establishment of the EP at P12 was unaffected in conditional Cx26 null mice, in which Cx26 was deleted from the support cells of the sensory epithelium, and also was unaffected in transgenic mice expressing the dominant-negative Cx26 mutant R75W (Cohen-Salmon et al., 2002; Kudo et al., 2003). In the latter study, the R75W mutant gene was introduced using Cre-loxP recombination under a CAG promoter, which has widespread activity and resulted in deafness associated with deformity of the supporting cells, loss of the tunnel of Corti and degeneration of sensory hair cells. EP does decline with Cx26 deficiency as postnatal development proceeds, likely due to loss of integrity of the epithelium. Interestingly, the deafness at birth caused by Cx26 deficiency is not associated with cell degeneration or a reduction in EP (Liang et al., 2012; Chen et al., 2014). Thus, alternatively, GJs in support cells likely play a developmental role that impacts on cochlear function early on (Wang et al., 2009) and by providing local K^+^ buffering and/or metabolic coupling that ultimately impacts on cell integrity. Since the organ of Corti is avascular, it was suggested that GJs between the support cells may help deliver glucose from the highly-vascularized stria vascularis (Chang et al., 2008). GJs also were reported to mediate the spread of Ca^2+^ waves in support cells of the organ of Corti through the intercellular diffusion of IP_3_ and subsequent Ca^2+^ release (Beltramello et al., 2005). Ca^2+^ acting on the Ca^2+^-dependent Cl–K co-transport systems may function to maintain the ionic balance of cochlear fluids.

Although Cx30 is co-localized with Cx26 and deletion of Cx30 produces deafness in mice, it is now apparent that the loss of Cx30 itself is not the cause of deafness. Cx26 and Cx30 appear to be co-regulated and expression of Cx26 protein is reduced in the cochlea of Cx30 null mice (Ahmad et al., 2007; Ortolano et al., 2008; Kamiya et al., 2014). When Cx26 expression was increased in Cx30 null mice by introducing a bacterial artificial chromosome containing additional copies of the Cx26 gene, hearing was rescued (Ahmad et al., 2007). The reciprocal experiment of increasing Cx30 expression in Cx26 null mice did not restore hearing (Qu et al., 2012). Furthermore, deletion of Cx30 using a strategy in which Cx26 expression was preserved did not result in hearing loss (Boulay et al., 2013). These results indicate that deafness in original reports of Cx30 KO mice were likely due to reduced expression of Cx26. Thus, although the specific roles of GJs in the cochlea have yet to be defined, it is apparent that expression of Cx26, but not Cx30, is crucial for auditory function. Interestingly, replacement of Cx26 with Cx32 under the Cx26 promoter resulted in mice with normal hearing (Degen et al., 2011) suggesting Cx32 and Cx26 are interchangeable, at least in terms of Cx26 loss-of-function models for deafness.

What about the function of Cx HCs in the cochlea? Dye uptake studies using multiple fluorescent probes reported increased uptake only in cochlear support cells that was ascribed to functional HCs (Zhao, 2005). Using isolated cochleae or explant cultures, studies have reported that HCs act as conduits for the release of ATP and inositol 1,4,5 triphosphate, IP_3_, from support cells (Zhao et al., 2005; Anselmi et al., 2008; Gossman and Zhao, 2008). There is Cx expression both in basolateral and apical regions of support cells, suggesting that signaling molecules could be released both into perilymphatic and endolymphatic compartments. Most studies have focused on ATP release. Using cochleae isolated form adult guinea pigs, ATP release into the endolymph was detected suggesting the possibility of direct modulation of outer hair cell (OHC) electromotility through activation of purinergic receptors (Zhao et al., 2005; Yu and Zhao, 2008). In mouse cochlea, ATP release through HCs was suggested to mediate Ca^2+^ wave propagation within the support cell network (Anselmi et al., 2008). The link between Ca^2+^ signaling, wave propagation and hearing may reside, in part, through effects on coordinated connexin expression in cochlear support cells (Rodriguez et al., 2012; Mammano, 2013). It is also possible that IP_3_ signaling modulates other components, such as K^+^ channels and co-transporters in the non-sensory epithelial cells and, through propagation, to the intermediate cells (IC) of the stria vascularis that regulates K^+^ flux into the intrastrial space and ultimately into marginal cells (MC) and the endolymphatic compartment. Developmentally, spontaneous release of ATP through HCs in support cells in the developing rat cochlea was suggested as a mechanism by which hair cells depolarize and synchronize their outputs to promote firing of the auditory nerve, which could help refine the development of central auditory pathways prior to the onset of hearing (Tritsch et al., 2007).

ATP and IP_3_ release from cochlear support cells was attributed to Cx HCs after ruling out other pathways such as P2X receptors, pannexin channels and anion channels through the use of blockers, although blocker specificity is lacking. In the case of ATP release via Cx HCs, both Cx26 and Cx30 presumably can serve this role. The presence of HC activity in support cells of the cochlea has been inferred from dye uptake, but has not been confirmed electrophysiologically. To date, no specific blockers have been identified for Cx HCs, although ATP release was blocked by La^3+^, which generally has been shown to block dye uptake attributed to open Cx HCs in other preparations (Sáez et al., 2010).

## Syndromic deafness mutations cluster in NT and E1 domains

The Cx subunits that constitute a HC have four transmembrane domains (TM1–TM4), with N-terminal (NT) and carboxy-terminal (CT) domains located on the cytoplasmic side (Figure 1C). The two extracellular loop domains, E1 and E2, each consisting of ~30–35 residues, mediate docking of the HCs to form GJ channels. An interesting feature that emerges from the mapping of the syndromic deafness mutations onto topology of a Cx subunit is that they largely cluster in the NT and E1 domains (Figure 1C). In contrast, the ~100 non-syndromic Cx26 deafness mutations (not shown) are scattered throughout the Cx protein (see Lee and White, 2009).

Although members of the Cx family can be classified into phylogenetic groups (Bennett et al., 1994; Cruciani and Mikalsen, 2007) there is notable sequence conservation in the transmembrane and extracellular domains and HCs and GJ channels formed of different Cxs share the same overall properties in that they permit the passage of hydrophilic dyes, they possess the same voltage gating mechanisms and respond to the same classes of inhibitors, e.g., acidification, long-chain alkanols, glycyrrhetinic acid and fenamates. Channel opening is controlled by voltage and there are two distinct gating mechanisms, both of which are properties of HCs, whether in docked or undocked configurations (Bukauskas et al., 1995; Trexler et al., 1996). One mechanism is characterized by gating transitions to a stable subconducting state, termed the residual conductance state, and is generally referred to as V*_j_* gating because it was the first mechanism described that gated GJ channels in response to the voltage difference between two cells or the transjunctional voltage, V*_j_* (Harris et al., 1981; Spray et al., 1981). The polarity of V*_j_* gating is Cx-dependent and can be of either sign (Verselis et al., 1994). The second voltage gating mechanism closes channels fully, with gating transitions comprised a series of transient subconductance states, which gives them the appearance of being slow when recorded at typical filtering frequencies. This mechanism is termed “loop gating” as evidence suggests closure involves conformational changes in the extracellular loops (Trexler et al., 1996; Tang et al., 2009; Verselis et al., 2009). V*_j_* and loop gating are also referred to as fast and slow gating, respectively, according to the kinetics of the gating transitions (Bukauskas and Verselis, 2004; Fasciani et al., 2013).

Biophysical and structural studies of HCs and GJ channels composed of several different connexins, e.g., Cx46, Cx50, Cx26 and the chimera Cx32*Cx43E1, have shown that the NT and E1 domains constitute the bulk of the aqueous pore and contain elements essential for voltage gating and regulation (Verselis et al., 1994; Zhou et al., 1997; Pfahnl and Dahl, 1998; Oh et al., 1999; Purnick et al., 2000; Kronengold et al., 2003; Maeda et al., 2009; Tang et al., 2009; Verselis et al., 2009; Sánchez et al., 2010). In fact, exchange of the NT halves (NT through CL) of Cx46 and Cx50 was shown to result in HCs in which the gating and unitary conductance properties remarkably correspond to the WT Cx constituting the N-terminal half of the protein (Kronengold et al., 2012). The co-segregation of biophysical properties with the NT half extends to Ca^2+^- and K^+^-dependent regulatory mechanisms in Cx46 and Cx50 HCs (Srinivas et al., 2006). Thus, syndromic deafness mutants map onto the domains that constitute the core elements that determine gating, permeability and regulatory characteristics of Cx HCs and GJ channels.

## KID syndrome and the “leaky” HC hypothesis

Cx HCs have large aqueous pores, which not only permits inorganic cations and anions to pass, but also larger molecules such as metabolites, second messengers and other active biomolecules. In addition to ATP, signaling molecules reported to permeate through HCs include glutamate, IP_3_ and Ca^2+^ (reviewed by Evans et al., 2006). Although the large pore of a Cx HC makes it a potentially important contributor to tissue function, it also makes it potentially harmful if activity is too high, which could run-down ionic gradients and allow entry/exit of molecules deleterious to cell function and survival.

**Figure 4:**
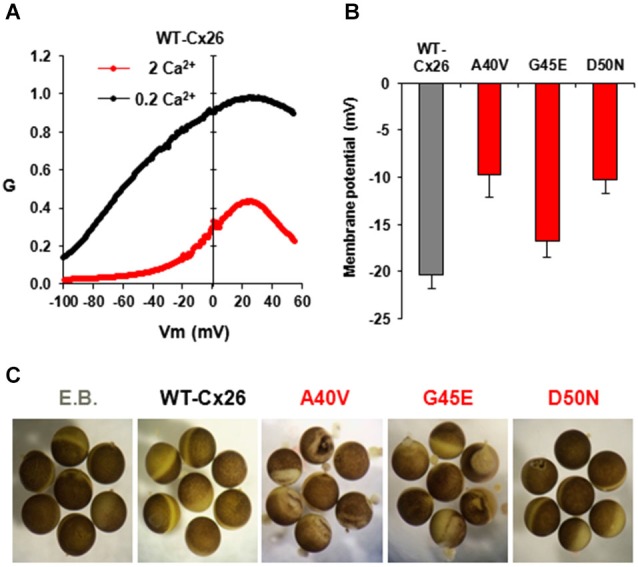
**HC activity in** ***Xenopus* oocytes injected with WT and mutant Cx26-mRNAs. (A)** Normalized G-V relationships of WT Cx26 HCs at two different external Ca^2+^ concentrations, 0.2 and 2 mM. Data were obtained by applying slow (600 s) voltage ramps from +60 to −100 mV from a holding potential of −20 mV. Ramps were obtained for each oocyte in 0.2 and 2 mM Ca^2+^ and normalized to the maximum value measured in 0.2 mM Ca^2+^. In WT Cx26, increasing Ca^2+^ shifted activation in the depolarizing direction and suppressed current magnitude. Adapted from (Sanchez et al., 2013). **(B)** Plot showing resting membrane potentials measured in oocytes 24–48 h after injection of RNA for WT-Cx26 and A40V, G45E and D50N mutants. Adapted from (Sánchez et al., 2010). **(C)** KID syndrome mutant HCs induce different degrees of cell damage in oocytes 4 days after RNA injection. As a control, oocytes were injected with elution buffer, E.B. (RNA vehicle). Oocytes expressing A40V, in particular, as well as G45E exhibit widespread disorganization of pigmentation and blebbing of the membrane. D50N exhibits delayed blebbing and cell death.

Undocked Cx HCs are closed at negative potentials by the loop-gating mechanism and opening is promoted by membrane depolarization. KID syndrome mutant HCs have been described as behaving in a “leaky” manner (Stong et al., 2006; Gerido et al., 2007; Lee et al., 2009; Mese et al., 2011; Mhaske et al., 2013). The “leaky” behavior broadly refers to increased HC activity, i.e., opening, in the membrane and has been attributed, almost solely, to impaired regulation of HCs by extracellular Ca^2+^. Extracellular Ca^2+^ shifts HC activation positive along the voltage axis (Ebihara and Steiner, 1993) and has been shown to act selectively on loop gating (Verselis and Srinivas, 2008). Thus, Ca^2+^ can act in conjunction with loop gating to effectively close Cx HCs over a wide range of voltages. In Cx26 HCs, V*_j_* gating operates only at larger inside positive voltages and is unlikely to play much of a role in gating Cx26 HCs. For loop gating, the shift in activation is substantial (Figure 4A) with V_1/2_, the voltage at which activation is half maximal, shifting ~50–60 mV with a 10-fold change in the extracellular Ca^2+^ concentration (Sánchez et al., 2010). Thus, normal plasma levels of Ca^2+^, 1–2 mM, would tend to keep Cx26 HCs closed at resting potentials with opening requiring a large depolarizing stimulus. At lower extracellular Ca^2+^ concentrations, less depolarization becomes necessary to elicit Cx26 HC opening, which can lead to substantial opening even at resting membrane potentials. Loss or weakened regulation by Ca^2+^ caused by KID mutations could increase Cx26 HC opening without a need for a reduction in extracellular Ca^2+^ levels. Since Cx26 HCs are not selective for Na^+^ and K^+^, increased opening of the mutant HCs would tend to collapse the resting membrane potential leading to cell dysfunction. Figure 4B shows the effects of expressing KID mutant A40V, G45E and D50N HCs on cell membrane potentials. After 48 h of expression in *Xenopus* oocytes, resting membrane potentials in oocytes expressing A40V and D50N were lower than those expressing WT Cx26; interestingly, G45E showed similar resting potentials to WT Cx26. After 4 days, the mutants tended to accelerate rundown of the membrane potential and to lead to cell death, although the extent to which this occurred differed among the mutants (Figure 4C).

Thus far, impaired inhibition by extracellular Ca^2+^ has been reported for G11E, G12R, N14K, A40V, G45E, D50N/A mutant Cx26 HCs (Gerido et al., 2007; Lee et al., 2009; Sánchez et al., 2010; Terrinoni et al., 2010; Sanchez et al., 2013). However, the degree to which inhibition by Ca^2+^ is impaired does not correlate with the severity of the disease phenotype. Thus, as we discuss in the following sections, other aberrant HC properties, other than simply increased activity due to impaired inhibition by Ca^2+^, are emerging as potential contributors to disease pathogenesis.

## KID mutant HCs: altered Ca^2+^ permeability

More extensive biophysical examination of three KID mutants, A40V, G45E and D50N, which are clustered near the TM1/E1 border and in the proximal segment of E1, revealed that two of the three residues, G45 and D50, are pore-lining (Sánchez et al., 2010; Sanchez et al., 2013) Unitary conductance of A40V HCs was indistinguishable from WT Cx26, whereas both G45E and D50N HCs showed substantially altered conductances (Sánchez et al., 2010). For G45E, unitary conductance was ~20% higher than WT Cx26 whereas for D50N it was ~50% lower and exhibited strong outward rectification of the open HC current. Furthermore, cysteine substitutions at these positions and subsequent application of thiol-modifying, methane-thiosulfonate reagents from either side of the membrane confirmed that G45 and D50, but not A40, are exposed to the aqueous pore in the open state of the Cx26 HC. Modification of G45C to a positively charged side chain with MTSET irreversibly reduced unitary conductance, whereas modification to a negatively charged side chain with MTSES had the opposite effect, increasing unitary conductance, much like the G45E KID mutation. Modification of D50C with oppositely charged reagents had the same qualitative effect, with MTSES essentially restoring WT conductance and rectification. Thus, charges at these positions strongly influence ion flux through the HC pore, explaining the effects of the KID mutations on HC conductance and rectification.

**Figure 5:**
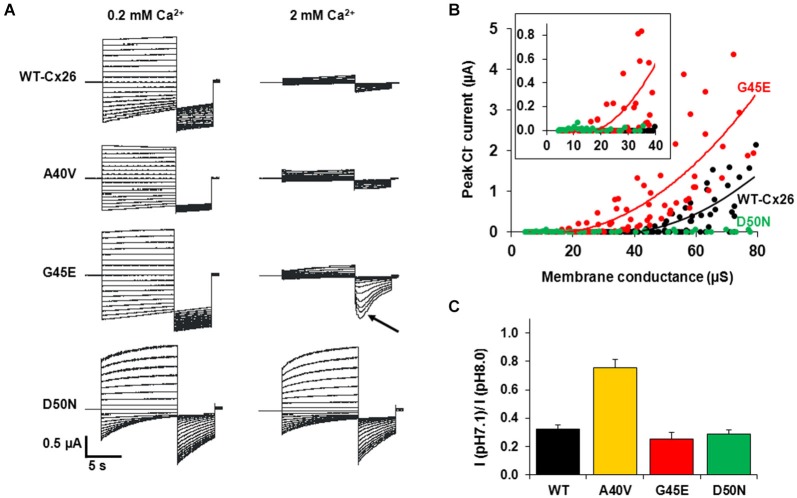
**KID syndrome mutations produce functional HCs, but with differential effects on regulation by Ca^2+^ and pH and permeability to Ca^2+^. (A)** Representative currents elicited by a series of voltage steps (10-s steps from +60 to −100 mV in intervals of 10 mV followed by a 5-s step to −110 mV) applied to oocytes expressing WT Cx26, A40V, G45E and D50N. Oocytes were voltage clamped to −20 mV. Currents shown in each case are from the same oocyte exposed to 0.2 and 2.0 mM Ca^2+^. WT Cx26, A40V, and G45E all showed substantial reductions in current magnitude in 2 mM Ca^2+^, whereas D50N was nearly insensitive to Ca^2+^. A large transient inward Ca^2+^-activated Cl current developed in oocytes expressing G45E when the voltage was stepped to −110 mV after depolarizing steps that activated these HCs (arrow). This was due to increased Ca^2+^ entry through G45E HCs. Data from (Sanchez et al., 2013). **(B)** Ca^2+^ activated Cl^−^ current plotted as a function of the macroscopic conductances of WT (black circles), G45E (red circles) and D50N (green circles) HCs in *Xenopus* oocytes. Solid lines correspond to regression lines fit to the data (best fit with a single exponential function). Inset: expanded view highlighting that only G45E induces Cl^−^ currents at considerably lower HC conductance values. Adapted from (Sánchez et al., 2010). **(C)** Bar graph showing the percent remaining HC current at pH 7.1 relative to a maximum at pH 8.0 (I_pH 7.1_/I_pH 8.0_). Currents were measured at a holding potential of −40 mV. Adapted from (Sanchez et al., 2014). Each bar represents the mean ratio ± SE. *n* = 5 for WT Cx26, 5 for A40V, 5 for G45E, and 5 for D50N.

A potentially significant impact of the G45E mutation was uncovered by Sánchez et al. (2010). As shown in Figure 5A, steps to positive membrane potentials in the presence of 2 mM extracellular Ca^2+^ in oocytes expressing WT or G45E HCs produced relatively small outward currents due to the robust inhibitory effect of Ca^2+^ on HC activation. For D50N HCs, however, outward currents in 2 mM extracellular Ca^2+^ were large due to loss of the inhibitory effect of Ca^2+^. However, if at the end of the activation step, the membrane was stepped to a large negative potential, the peak tail current for G45E was followed by activation of a large inward current. Using ion substitutions, blockers and intracellular chelation of Ca^2+^, this current was established to be a Ca^2+^-activated Cl current endogenous to *Xenopus* oocytes. Thus, it appeared that Ca^2+^ entry through G45E HCs was activating the Cl channel. Examination of the relationship between the Cl current and HC activation showed that substantially fewer G45E HCs were needed to activate Cl currents than WT Cx26 HC (Figure 5B). D50N HCs failed to activate Cl currents even with many HCs activated. These data demonstrate that the G45E KID mutation substantially increases permeability of Cx26 HCs to Ca^2+^. Conversely, D50N appears to greatly diminish or abolish Ca^2+^ permeability.

## Other potential HC regulators in KID syndrome

Cx HCs are not only regulated by extracellular Ca^2+^, but also by other divalent cations, including Zn^2+^, as well as pH (Ebihara and Steiner, 1993; Trexler et al., 1999; Chappell et al., 2004; Ripps et al., 2004). For Cx26 HCs, sensitivity to pH falls in a physiological range such that substantial inhibition occurs at normal plasma pH levels of ~7.4. Examination of KID syndrome mutant HCs shows a reduced sensitivity to pH for A40V (Sanchez et al., 2014). Figure 5C shows that a change in extracellular pH from 8.0 to 7.1 produces substantial inhibition of WT Cx26 HCs, reducing current ~70%. However, this inhibition is substantially less for A40V, ~25%. For comparison, G45E and D50N mutant HCs show the same responses to pH as WT Cx26 HCs. Thus, among these three mutants, pH sensitivity is only affected in A40V. Interestingly, A40V also selectively showed a reduction in sensitivity to Zn^2+^ (Sanchez et al., 2014). Thus, the A40V substitution appears to exert its effects mainly through increased opening resulting from alterations in a multiplicity of HC regulatory mechanisms.

A hallmark of patients afflicted with syndromic deafness due to mutations in Cx26 is susceptibility to skin infections and neoplasms that can lead to squamous cell carcinomas (Coggshall et al., 2013). Examination of the effects of peptidoglycans from *Staphylococcus aureus*, an opportunistic pathogen, on a keratinocyte cell line, HaCaT, showed induced ATP release attributed to increased Cx26 HC activity (Donnelly et al., 2012). Moreover, ATP release was significantly higher in cells expressing KID mutants suggesting these mutant HCs trigger pro-inflammatory events in response to peptidoglycans from opportunistic pathogens.

Several studies have indicated opening of HCs can be induced by metabolic inhibition (John et al., 1999; Contreras et al., 2002; Vergara et al., 2003; Sánchez et al., 2009). Induced HC opening with metabolic inhibition occurs without changes in extracellular Ca^2+^. The mechanism by which HCs are opened is not understood, but possibilities include depletion of ATP leading to increased intracellular Ca^2+^ and possibly altered Cx phosphorylation. Interestingly, intracellular Ca^2+^ has been shown to modulate HC opening (De Vuyst et al., 2006, 2009; Schalper et al., 2008). The response of HCs to intracellular Ca^2+^ is bell-shaped, with opening increasing up to ~500 nM, and deceasing with larger Ca^2+^ concentrations (De Vuyst et al., 2009).

HC regulatory mechanisms, whether related to intracellular Ca^2+^, peptidoglycans or to pathways involving ATP depletion, changes in kinase activity or free radical generation indicate that HC opening can be regulated in many ways and Cx mutations can affect any one of these processes. At this point, effects of syndromic deafness mutations on HC opening under metabolic stress or ischemia have not been evaluated.

## Genotype-phenotype relations

D50N HCs exhibit a near loss of inhibition by Ca^2+^, but hearing loss in patients carrying this mutation can be moderate to severe with serious, although not fatal, cutaneous manifestations (Richard et al., 2002; van Steensel et al., 2002; Yotsumoto et al., 2003). On the other hand, G45E HCs exhibit near normal inhibition by Ca^2+^, but this mutant leads to profound deafness and causes a lethal form of KID syndrome with fatality often occurring in the first year of life due to uncontrollable skin infections (Janecke et al., 2005; Jonard et al., 2008; Sbidian et al., 2010).

The finding that G45E HCs exhibit increased permeability to Ca^2+^ (Sánchez et al., 2010) provides a compelling argument for the severity of the phenotype in patients carrying this mutation. Increased Ca^2+^ entry, even with modest HC activity, could trigger Ca^2+^-induced cascades that lead to broad dysfunction and/or cell death. An autopsy of a patient carrying the G45E mutation showed widespread vestibulo-cochlear dysplasia in the organ of Corti with no evidence of a developed sensory epithelium (Griffith et al., 2006). Thus, the G45E mutation, indeed, leads to devastating effects in the organ of Corti. In patients carrying the D50N mutation, the cochlea appears to develop normally, and hearing loss is less profound. Although D50N HCs are not appreciably inhibited by Ca^2+^, which by inference should produce a severe phenotype, these HCs also show a gating phenotype characterized by a substantial shift in activation in the hyperpolarizing direction (Lopez et al., 2013; Sanchez et al., 2013). Thus, HC opening is reduced even when resting potentials are modest, thereby potentially mitigating the loss of inhibition by Ca^2+^. This scenario would be particularly applicable to cochlear support cells that typically maintain robust resting potentials of ~−80 mV (Lang et al., 2007). D50N also shows no or greatly reduced Ca^2+^ permeability, which also may help mitigate the loss of inhibition by Ca^2+^. A40V is not a pore-lining mutation and shows no evidence that it alters HC conductance and permeability (Sánchez et al., 2010). However, A40V exhibits a multiplicity of effects including impaired inhibition by Ca^2+^, pH and Zn^2+^ (Sanchez et al., 2014). The combined impairment of these regulatory mechanisms may be responsible for the severe A40V phenotype that would not be predicted from impaired Ca^2+^ inhibition alone.

Although combinations of altered biophysical properties may explain the phenotypic diversity among KID mutants, increased HC activity due to more efficient expression and/or trafficking to the membrane must also be considered. Certainly, in exogenous expression systems, currents produced by some mutant HCs are larger even when protein expression levels are taken into account (Gerido et al., 2007). Whether this increased expression of KID mutants holds true in native tissue remains to be determined. In skin, Cx26 plays a key role in epidermal development and wound healing (Lucke et al., 1999; Coutinho et al., 2003; Richard, 2005; Djalilian et al., 2006). Persistent expression of Cx26 has been shown to maintain wounded epidermis in a hyperproliferative state and to block remodeling leading to infiltration of immune cells (Djalilian et al., 2006). Furthermore, ectopic Cx26 expression was shown to increase ATP release, resulting in delayed recovery of the epidermal barrier and promotion of an inflammatory response. However, overexpression of WT Cx26 leads to psoriatic skin and not the more severe phenotypes characteristic of KID syndrome. Thus, increased expression of Cx26 HCs alone does not appear to be sufficient to cause KID syndrome suggesting that aberrant biophysical properties are important. Even with a small sample of three KID mutants, genotype-phenotype relationships are emerging. Further biophysical examination of other KID mutants and syndromic should continue to shed light on the roles of HCs in cochlea in normal and pathologic conditions.

## Conflict of interest statement

The authors declare that the research was conducted in the absence of any commercial or financial relationships that could be construed as a potential conflict of interest.
